# Simultaneous determination of epinephrine and folic acid using MIL-101 (Fe)-NH_2_ metal-organic framework/graphene oxide nanocomposite modified electrode

**DOI:** 10.5599/admet.2762

**Published:** 2025-06-19

**Authors:** Rasha Kareem Khundhur

**Affiliations:** Pharmacology Department, College of Medicine, University of Misan, Misan, Iraq

**Keywords:** Chemically modified electrode, real sample analysis, pharmaceutical preparations

## Abstract

**Background and purpose:**

It is generally known that the majority of disorders exhibit symptoms to some degree when the quantities of two crucial substances, epinephrine and folic acid, are low or high. These two chemicals' composition variations may be tracked and utilized to identify conditions such as myocardial infarction, Parkinson's disease, and mental disorders.

**Experimental approach:**

Using a solvothermal technique, we propose the synthesis of a novel MIL-101 (Fe)-NH_2_ metal-organic framework/graphene oxide nanocomposite (MOF/GO nanocomposite). The produced nanocomposite's morphology was examined using field-emission scanning electron microscopy. A straightforward, quick, and sensitive electrochemical sensing platform for epinephrine detection was then created by drop-casting the produced MOF/GO nanocomposite onto the screen-printed electrode (SPE).

**Key results:**

Compared to unmodified SPE, cyclic voltammetry revealed that the MOF/GO/SPE considerably enhanced the epinephrine oxidation process, exhibiting a greater detection current at a lower over-potential. The synergistic combination of MOF and GO sheets may cause this discovery. With a low detection limit of 0.07 μM, the MOF/GO/SPE sensor's linear response for voltammetric measurements of epinephrine was found to be between 0.2 and 500.0 μM. A modified electrode was also utilized to measure folic acid and epinephrine simultaneously.

**Conclusion:**

Lastly, the modified SPE effectively demonstrates its high accuracy in identifying folic acid and epinephrine in biological and pharmaceutical samples.

## Introduction

Chemical sensors and biosensors have attracted a lot of attention in modern analytical chemistry fields because of their excellent sensitivity and specificity [1,2]. Electrochemical sensors have overtaken optical, piezoelectric, thermometric, and magnetic sensors as the most widely used sensor types on the market because of their low cost, simplicity, ease of automation, and decreasing capabilities [3].

The catecholamine neurotransmitter epinephrine (EPI) (1-(3,4-dihydroxyphenyl)-2-methylaminoethanol), often known as adrenaline, is necessary for information transfer between biological cells in the mammalian central nervous system [4,5]. It is a large organic cation found in bodily fluids and nervous tissue. It has a major impact on how the cardiovascular, hormonal, and central neurological systems operate. The level of EPI in blood is linked to a number of biological events. Numerous disorders are indicated by changes in their blood concentration [6]. In medicine, EPI is frequently used as an emergency drug [6]. This catecholamine drug is used to treat cardiac surgery, sepsis, bronchial asthma, myocardial infarction, and hypertension [5,6]. Because EPI is both a powerful doping agent and a medicinal treatment, the World Anti Doping Agency (WADA) prohibits its usage during athletic competitions [7]. Therefore, figuring out how much EPI is in body fluids has drawn a lot of attention from academics [7].

A water-soluble B-vitamin, folic acid (FOA), is found naturally in many foods, including fruit, almonds, broccoli, cabbage, and cauliflower. A lack of FOA in the diet is strongly associated with neural tube abnormalities in infants as well as a higher risk of megaloblastic anaemia, cancer, Alzheimer's disease, heart disease, and some mental illnesses [8]. As a result, determining the amount of FOA in food, pharmaceutical, and clinical samples has received a lot of attention, and a sensitive and trustworthy detection technique is much anticipated [9]. FOA has been detected using electrochemical techniques. Additionally, a great deal of attention has been generated by its benefits, which include simplicity, quick reaction, high stability, outstanding repeatability, low cost, and low detection limit [10,11]. The development of electrochemical techniques for the measurement of EPI and FOA in blood serum and urine has drawn a lot of interest in recent years. Compared to other detection methods, electrochemical detection is quick, sensitive, environmentally friendly, and precise. However, due to the two biomolecules' too-close oxidation potentials and low sensitivity at the bare electrode, the simultaneous measurement of EP and FOA has seldom ever been reported [12,13].

One of the leading challenges in the domain of electrode modification is crafting catalysts that can endure a great deal of electrochemical activity with little to no deterioration of properties, which can stimulate the switching of the oxidation potential and voltammetric responses of EPI and FOA. Lately, a range of chemically modified electrodes (CMEs) have been discovered for the simultaneous detection of EPI and FOA [13]. Given the many special features that the metal-organic frameworks (MOFs) possess, they have been the ideal candidate as the supporting material to achieve electrode modification [14].

In the past few years, metal-organic frameworks have been a subject of interest to numerous scientists, who have understood that it is possible to use them in various fields of science. These porous crystalline materials are made of organic linkers and metal ions. The power to control matter from the atomic and molecular (nanoscale) world is particularly important in the development of metal-organic frameworks. It is in this light that we should consider MOFs and organic linkers as an excellent starting point since they can be used in the fields of biomedicine, biomedical applications, and sensing applications [14,15]. Of course, there are a handful of unique structural features, which in fact, make these highly crystalline materials unobtainable through standard methodologies.

To make MOFs more conducive, stable, and more catalytically active, they can be easily either combined or, in order to be stabilized, supplemented with various functional materials [16]. The special properties of these compositions are that they are different from pure MOFs. Graphene oxide (GO), made up of oxygen functional groups attached to its surface, can offer good electrochemical performance in various fields due to its high active surface area, chemical stability, and adjustable functional groups. This carbon-based material has been applied to making biosensors, supercapacitors, as well as electrochemical sensors [17]. The superior potential of GO properties can be harnessed for fuel cells, next-generation batteries, and catalysis, in which energy storage and sensing play a role [18].

One well-established technique that finds appropriate use in the mass manufacturing of electrodes (SPEs) for electrochemical sensors and biosensors is screen-printing technology. This technology's adaptability, affordability, mass production potential, and capacity to create bespoke electrode combinations with various substrates, forms, geometries, and other features are among its many noteworthy benefits for building SPEs. Because of their advantages over traditional sensors, such as their small size, mobility, simplicity of manufacture, and affordability, screen-printed carbon electrodes (SPCEs) have found an appropriate and successful place in the electroanalysis of a variety of chemicals in recent years. Because of their extremely narrow working area, SPCEs are ideal for detecting traces of target species. They have excellent sensitivity, are dependable, and are simple to use [19]. A significant overvoltage is required for the rapid electrochemical reaction of different analytes on the surface of the bare electrodes. Adding a suitable surface modification to the electrode can be a useful way to deal with these problems. The electron transport between the electrodes and the electroactive species can be improved with the right modifier. The development of modified electrodes with superior electro-catalytic qualities and enhanced sensitivity for target analyte detection has received attention recently [20].

In this paper, we describe an innovative and highly efficient sensing platform that relies on a nanocomposite of MOF and GO with the aim of improving the EPI determination. The MOF/GO/SPCE sensor platform was much better than the unmodified SPCE electrochemically in the level determination of EPI due to its larger active surface area. In line with the quantitative study, the recommended sensor proved highly sensitive for EPI detection, presenting a low detection limit (LOD) coupled with high selectivity over a wide linear detection range.

## Experimental

### Reagents and apparatus

Sigma-Aldrich was the source of EPI, FOA, methanol, phosphoric acid, sodium hydroxide, 2-aminoterephthalic acid, FeCl_3_.6H_2_O, N,N-dimethylformamide (DMF), graphene oxide, and all other chemicals. The Netherlands' μ-Autolab system using NOVA software was used for electrochemical tests. All the electrochemical tests were conducted using the SPCEs (DRP-110 model-Dropsens, Spain). A carbon working electrode, a carbon counter electrode, and an Ag pseudo-reference electrode make up each SPCE's three-electrode system.

### Synthesis of metal-organic framework/graphene oxide nanocomposite

The solvothermal synthesis of MOF/GO nanocomposite was carried out, with minor modifications, based on an earlier study [21]. To do this, 40 mL of N,N-dimethylformamide (DMF) was mixed with 2.48 mmol of the 2-aminoterephthalic acid (0.448 g) and 4.96 mmol of FeCl_3_.6H_2_O (1.34 g) and stirred magnetically for 10 minutes at room temperature. After that, 0.012 g of GO was added to the solution above and ultrasonically agitated for 40 minutes. Following ultrasonication, this suspension was put into a stainless-steel autoclave lined with Teflon (polytetrafluoroethylene) and heated to 110 °C for 24 hours in an oven to undergo a solvothermal reaction. The autoclave was then removed from the oven and allowed to cool to room temperature. Following cooling, centrifugation was used to collect the produced precipitate, which was then repeatedly cleaned with ethanol and DMF. Lastly, the product was dried for 15 hours at 65 °C in a vacuum oven.

### Modification of screen-printed carbon electrode

Electrochemical studies were conducted using the modified SPCE of the MOF/GO nanocomposite. Consequently, 1 mg mL^-1^ of MOF/GO suspension was initially made in water by ultrasonication for 20 minutes to modify SPCE. The SPCE surface was then drop-cast with 4.0 μL of MOF/GO ultrasonicated suspension. It was then dried for 20 minutes at room temperature.

### Real sample preparation

Urine samples were collected and quickly stored in a refrigerator. The materials were centrifuged in 10 mL for 15 minutes at 2000 rpm. To extract the supernatant, a 0.45 μm filter was utilized. Different amounts were then added to a 20 ml volumetric flask, and the mixture was diluted to the proper level with 0.1 M PBS (pH 7.0). Different quantities of FOA and EPI were added to the diluted urine samples. To determine the amounts of FOA and EPI, the recommended technique employed the standard addition method.

A FOA tablet (5 mg *per* tablet) and an EPI injection (labelled value EPI = 1 mg *per* injection) were purchased from the local store. The 0.1 M stock solution of the EPI injection, prepared using water, was limited to 10 mL. The electrochemical cell with a 10 mL 0.1 M PBS (pH 7) solution was filled with the required volume of this solution for the detection of EPI. The FOA pills were carefully ground and homogenized, and then 10 mL of the 0.1 M stock solutions of the FOA were prepared. Sonicating was carried out just before filtering to ensure that solid particles were completely dissolved. The electrochemical cell was set for analysis with the clean filtrate of 10 mL of PBS as the required volume after filtration.

## Results and discussion

### Metal-organic framework/graphene oxide nanocomposite characterization

**Figure 1.**
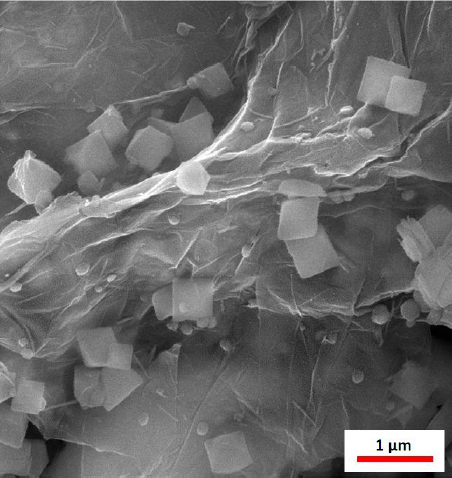
MOF/GO nanocomposite FE-SEM image

Field-emission scanning electron microscopy (FE-SEM) images helped in analysing the morphology and structures of the MOF/GO nanocomposite. The FE-SEM images are presented in Figure 1. The FE-SEM images reveal the morphology and structure of the MOF with an octahedral configuration, together with the GO sheets. The FE-SEM images very unequivocally identify the two components, GO and MIL-101(Fe)-NH_2_ MOF, present in the synthesized nanocomposites. Furthermore, the detected octahedral shape of MOF was consistent with previous publications.

### Electrochemical behaviour of epinephrine at the surface of the metal-organic framework/graphene oxide screen-printed electrode

**Figure 2.**
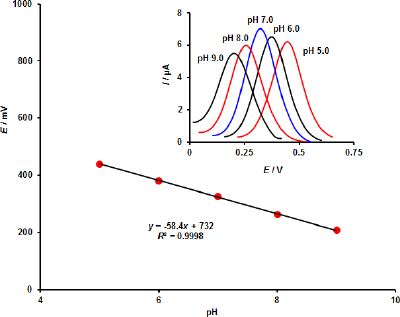
The current-pH curve and corresponding DPVs (inset) for electro-oxidation of 70.0 μM EPI at MOF/GO/SPE at a scan rate of 50 mV s^–1^

Using differential pulse voltammetry (DPV) at the MOF/GO/SPE surface, we examined the electrochemical behaviour of EPI in the pH range of 5.0 to 9.0 (inset Figure 2). Figure 2 gives a regression equation, *E*_p_ = -58.4*x* + 732 (*R*^2^ = 0.9998), which verifies that the electrooxidation of EPI involves an equal amount of protons and electrons. Furthermore, the optimal electrooxidation setting for the subsequent study was determined to be at pH 7.0, where the highest oxidation peak current was measured. The Randles-Ševčik equation was used to calculate the active surface area of MOF/GO/SPE and bare SPE in a 2 mM Fe(CN)_6_^3–/4–^ solution with 0.1 M KCl acting as a supporting electrolyte. The resulting surface areas for MOF/GO/SPE and bare SPE were 4.71 and 3.14 mm^2^, respectively.

**Figure 3.**
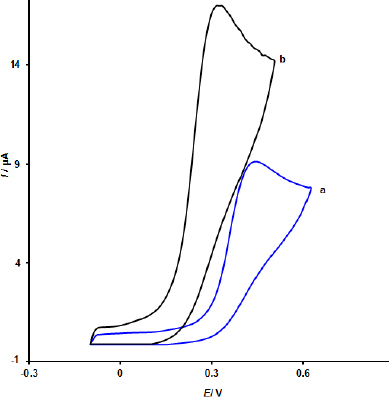
Bare SPE (a) and MOF/GO/SPE (b) cyclic voltammograms at pH 7.0 with 200.0 μM EPI present, respectively

The cyclic voltammetry curves of MOF/GO/SPE and bare SPE (curve a) at pH 7.0 with 200.0 μM EPI are shown in Figure 3. An irreversible signal with a peak potential of around 450 mV and a modest oxidation current of about 9.5 μA was produced on a bare SPE. Following MOF/GO electrode modification, the peak current rose to approximately 17.0 μA. The catalytic effect of MOF/GO may be the reason why the peak current in the MOF/GO /SPE scenario (curve b) was greater than that for the bare SPE (curve a).

**Figure 4.**
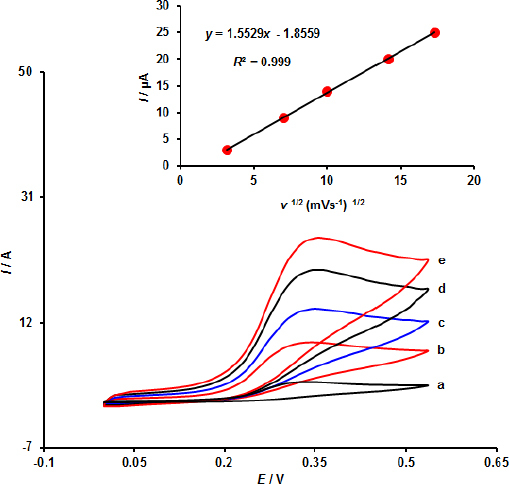
Cyclic voltammograms of 100.0 μM EPI at MOF/GO/SPE at various scan rates: a-e of 10, 50, 100, 200 and 300 mV s^–1^. *I*_pa_ *vs. v*^1/2^ plot for EPI oxidation at MOF/GO/SPE is displayed in the inset

According to the scan rate analysis, the oxidation current of EPI in the range under study (*i.e.* 10 to 300 mV s^–1^) was linear with *v*^1/2^ (Figure 4). The electrochemical oxidation of EPI at the MOF/GO/SPE surface is diffusion-controlled, according to the relationship between *I* and *ν*^1/2^.

**Figure 5.**
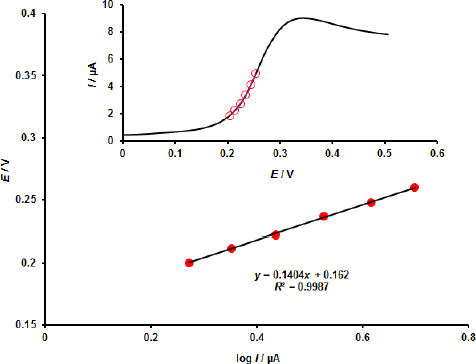
MOF/GO/SPE Tafel plot with 100.0 μM EPI and the corresponding linear sweep voltammograms

Furthermore, the Tafel plot of log *I* - *E* is linear and has the regression equations log *I* = 0.1404 *E* + 0.162, with *I* in μA, *E* in V, and *R*^2^ = 0.9987 (Figure 5). The value of α was determined to be around 0.58.

**Figure 6.**
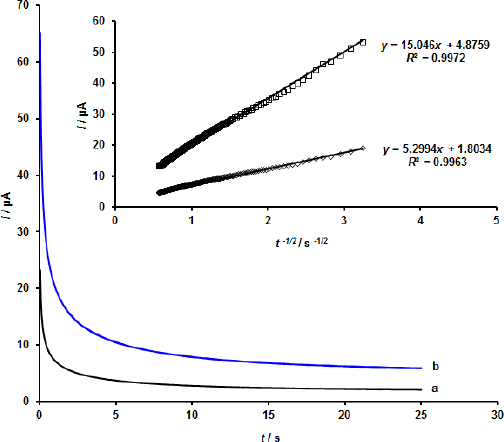
Chronoamperograms at MOF/GO/SPE with different EPI concentrations at pH 7.0 and Cottrell’s visualization of the chronoamperogram data

In the current study, the diffusion coefficient (*D*) of EPI was calculated using the chronoamperometric technique using the Cottrell equation (Figure 6). Using the Cottrell plots and Cottrell equations, the mean diffusion coefficient value was determined to be 3.04×10^–5^ cm^2^ s^–1^.

The construction of a new analytical sensor necessitates the presence of both long-term stability and repeatability. The four MOF/GO/SPE systems' repeatability was tested using 100.0 μM EPI solution, the resulting current response having a 3.5 % relative standard deviation (RSD). The long-term stability of the MOF/GO/SPE systems was monitored using the DPV technique within a 30-day interval with check-in periods of 10 days. The response current in the first 10 days reduced by 1.4 %, in the subsequent 10 days, it decreased by 2.6 %, and finally, in the last 10 days, it fell by 3.7 %. This clearly implies that the MOF/GO/SPE systems exhibit good long-term stability in the tests.

There were various solutions of EPI and FOA with different concentrations that were examined in optimized DPV research conditions. The regressions equations *I* = 0.0792*C* + 1.3074, *R*^2^ = 0.9998 and *I* = 0.069*C* + + 1.4693 were developed based on the calibration curves, and the signal of both combinations of the analytes EP and FOA was a linear function over the range of 0.2 to 500.0 and 0.5 to 600.0 μM, respectively. The estimated detection limit for EPI was 0.07 μM and for FOA 0.17 μM (*S*/*N* = 3).

**Figure 7.**
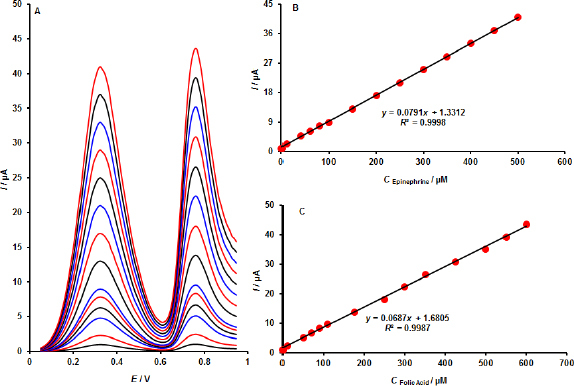
(A) MOF/GO/SPE DPVs in pH 7.0 with varying EPI and FOA concentrations (from inner to outers) with mixed solution of 0.2 + 0.5; 10.0 + 10.0; 40.0 + 50.0; 60.0 + 70.0; 80.0 + 90.0; 100.0 + 110.0; 150.0 + 175.0; 200.0 + 250.0; 250.0 + 300.0; 350.0 + 425.0; 400.0 + 500.0; 450.0 + 550.0 and 500.0 + 600.0 μM EPI and folic acid, respectively. Plots B and C show the peak currents in relation to the concentrations of EPI and FOA, respectively

By altering the concentrations of FOA and EPI at the same time, the use of the MOF/GO/SPE in the simultaneous measurement of these two substances was examined (Figure 7A). Two distinct and well-defined oxidation peaks were visible in the DPV data at potentials of 320 and 760 mV, corresponding to the EPI and FOA, respectively. Conversely, when EPI and FOA concentrations were increased concurrently, the peak currents of both substances linearly increased, with sensitivity values of 0.0791 and 0.0687 μA μM^–1^ (Figures 7B, C). The EPI sensitivities in the presence and absence of FOA are almost identical, indicating that EPI can be determined in the presence of FOA.

The influence of possible interfering species was also determined. The determination had a relative error of about ±5 % since the tolerance limit was interpreted as the highest concentration of the interferents. 10^–1^ M for Cl^–^ and Na+, K^+^; 5.0×10^–2^ M for Ca^2+^ and Mg^2+^; 4.0×10^–3^ M for NADH, l-asparagines, glucose, glutamic acid, acetaminophen, l-cysteine, l-cystine, glycine, and l-lysine, were the acceptable concentrations of foreign chemicals.

The suggested approach was also used to determine the levels of FOA and EPI in oral FOA tablets and EPI injections to assess their analytical applicability. To determine the quantities of EPI and FOA in the pharmaceutical preparations, DPV responses of the diluted analyte and the sample that was spiked with a specific quantity of EPI and FOA were used. Table 1 presents the findings. By contrasting the acquired results with those stated on the label of the medicinal preparations, the suggested modified electrodes' dependability was also assessed (Table 2). RSD and acceptable recovery rates for the spiked sample are displayed in Table 2.

**Table 1.** Determination of EPI and FOA in real pharmaceutical preparations using the MOF/GO/SPE (*n* = 5)

Sample	Amount, μM				Recovery, %		RSD, %	
	Spiked		Found					
	EPI	FOA	EPI	FOA	EPI	FOA	EPI	FOA
EPI injection	0.0	0.0	2.9	-	-	-	3.3	
	2.0	5.0	5.0	4.9	102.0	98.0	2.9	3.5
	4.0	7.0	6.7	7.3	97.1	104.3	2.1	2.2
FOAablet	0.0	0.0	-	4.9	-	-	-	-
	5.5	2.0	5.4	7.1	98.2	102.9	2.3	2.6
	7.5	4.0	7.6	8.8	102.7	98.9	2.8	3.4

**Table 2:** Comparisons of the total value of EPI and FOA of some pharmaceutical preparations obtained using MOF/GO/SPE with the declared value in the sample labels (*n*=5).

Samples	Declared	Found	RSD, %
EPI injection, mg ml^-1^	1.0	0.995	2.5
FOA tablet (mg *per* tablet)	5.0	5.045	3.0

Additionally, Table 3's statistics show that the outcomes of using MOF/GO/SPE are consistent with the statements made on the preparations' labels. As a result, the modified electrode may be effectively employed to determine FOA and EPI levels in medicinal formulations either separately or together.

**Table 3.** Determination of EPI and FOA in urine sample using the MOF/GO/SPE (*n* = 5)

Amount, μM				Recovery, %		RSD, %	
Spiked		Found					
EPI	FOA	EPI	FOA	EPI	FOA	EPI	FOA
5.0	5.5	5.1	5.3	102.0	96.4	2.2	3.0
7.0	7.5	6.9	7.6	98.6	101.3	3.6	2.5

The suggested approach was also used to determine the levels of FOA and EPI in urine samples to assess their analytical applicability. Table 3 presents the findings for identifying the two species in urine samples. FOA and EPI showed satisfactory recovery of the trial outcomes. The mean relative standard deviation (RSD) showed that the procedure was reproducible.

## Conclusion

A new voltammetric sensor for measuring EPI in the presence of FOA was created using the MOF/GO to create the MOF/GO/SPE system. The simultaneous measurement of FOA and EPI yielded low limits of detection. The voltammetric signals of FOA and EPI were satisfactorily resolved by the MOF/GO/SPE, with a peak separation of 440 mV. As the first electrochemical sensor to analyse FOA and EPI simultaneously in actual samples, the MOF/GO/SPE performs well.
